# Comparison of the clinical outcomes of laparoscopic-assisted versus open surgery for colorectal cancer

**DOI:** 10.3892/ol.2014.1859

**Published:** 2014-02-07

**Authors:** KAI CHEN, ZHUQING ZHANG, YUNFEI ZUO, SHUANGYI REN

**Affiliations:** 1 Department of General Surgery, The Second Hospital of Dalian Medical University, Dalian, Liaoning 116023, P.R. China; 2 Department of Clinical Biochemistry, College of Laboratory Diagnostic Medicine, Dalian Medical University, Dalian, Liaoning 116044, P.R. China

**Keywords:** clinical outcomes, colorectal cancer, laparoscopy, surgery

## Abstract

The present study aimed to compare the clinical outcomes of laparoscopic-assisted surgery versus open surgery for colorectal cancer and investigate the oncological safety and potential advantages and disadvantages of laparoscopic-assisted surgery for colorectal cancer. The medical records from a total of 160 patients who underwent surgery for colorectal cancer between January 2009 and January 2013 at The Second Hospital of Dalian Medical University (Dalian, China) were retrospectively analyzed. The patients who underwent laparoscopic-assisted surgery showed significant advantages due to the minimally invasive nature of the surgery compared with those who underwent open surgery, namely, less blood loss (P=0.002), shorter time to flatus (P<0.001), bowel movement (P=0.009) and liquid diet intake (P=0.015), earlier ambulation time (P=0.006), smaller length of incision (P<0.001) and a shorter post-operative hospital stay (P=0.007). However, laparoscopic-assisted surgery for colorectal cancer resulted in a longer operative time (P=0.015) and higher surgery expenditure (P=0.003) and total hospitalization costs (P<0.001) compared with open surgery. There were no statistically significant differences between the intraoperative and post-operative complications. There were no differences in the local recurrence (P=0.699) or distant metastasis (P=0.699) rates. In addition, no differences were found in overall survival (P=0.894) and disease-free survival (P=0.701). These findings indicated that laparoscopic-assisted surgery for colorectal cancer had the clear advantages of a minimally invasive surgery and relative disadvantages, including a longer surgery time and higher cost, and exhibited similar rates of recurrence and survival compared with open surgery.

## Introduction

Colorectal cancer is one of the leading causes of mortality worldwide, however, due to the development of minimally invasive techniques, the majority of colorectal procedures can also be performed using a laparoscopic approach, and the indications for laparoscopic-assisted surgery have gradually expanded (1,2). A number of available prospectively randomized trials and meta-analyses of laparoscopic-assisted surgery for colorectal cancer (3–8) reported that laparoscopic-assisted colorectal surgery exhibited improved post-operative results, including less pain, a smaller incision, a faster recovery of gastrointestinal function, a shorter post-operative hospital stay and similar long-term survival, compared with those of open colorectal surgery (9–13). Therefore, laparoscopic-assisted surgery has been widely accepted as an alternative to conventional open surgery for colorectal cancer (14).

Despite the theoretical advantages of laparoscopic-assisted surgery, it is not considered the standard surgical treatment for colorectal cancer due to criticism concerning its oncological stability (9,15). The potential risks include port-site recurrence following curative resection of the tumor and incomplete lymph node dissection. The present study aimed to compare the clinical outcomes of laparoscopic-assisted surgery versus open surgery for colorectal cancer and investigate the oncological safety and potential advantages and disadvantages of laparoscopic-assisted surgery for colorectal cancer.

## Materials and methods

### Patients

The medical records of a total of 160 patients who underwent surgery for tumor node metastasis (TNM) (16,17) stage I-IIIC colorectal cancer between January 2009 and January 2013 at The Second Hospital of Dalian Medical University (Dalian, China) were retrospectively analyzed. The medical records consisted of 80 cases of laparoscopic-assisted surgery (the laparoscopic group) and 80 cases of traditional open surgery (the open surgery group). Patients were non-randomized, enrolled and allocated to laparoscopic or conventional open surgery groups at the patients discretion. The inclusion criteria were as follows: All patients were diagnosed with colorectal cancer by pre-operative colonoscopy and biopsy analysis. All patients who were confirmed with colorectal cancer by physical examination [lung X-rays, pre-operative upper abdominal ultrasonography and abdominal computed tomography (CT)] exhibited no bowel obstruction or tumor invasion of the surrounding adjacent or distant organs. The exclusion criteria were as follows: Patients who required emergency surgery due to serious complications, including acute colorectal cancer obstruction or cancer perforation, cases with a history of pre-operative chemoradiotherapy and major abdominal surgery, cases with a previous history of abdominal surgery and cases in which a curative resection could not be performed. Data were collected and reviewed retrospectively, including patient demographics, pre-operative clinical characteristics, surgical procedures, pathological parameters, perioperative recovery and complications. This study was approved by the Research Ethics Committee of Dalian Medical University, and informed consent was obtained from all participants.

### Surgical technique

All surgeries were performed by the same team of surgeons who had proven expertise in colorectal cancer procedures and who perform >100 laparoscopic and open colorectal surgeries annually. All patients received cefminox (2.0 g) intravenously at the induction of general anesthesia for systemic antibiotic prophylaxis. Additional pre-operative preparations were standardized, following the course of traditional abdominal surgeries. For conventional open surgery, the patients were placed in the supine position or modified lithotomy position, and a midline or right paramedian skin incision was performed. Open procedures were performed according to standard techniques, which were applied by the operating surgeon. For laparoscopic-assisted surgery, the patient was placed in the modified lithotomy (supine) and trendelenburg positions. A pneumoperitoneum was created by the open method, and the CO_2_ pneumoperitoneum pressure was set at 12–15 mmHg. In this study, five ports were used: An umbilical port for the laparoscopic camera (CV180, Olympus Corporation, Tokyo, Japan) and two ports each in the right and left sides. For a right hemicolectomy, the surgeon and camera operator stood to the left side of the patient, and for a left hemicolectomy, the surgeon and camera operator stood to the right side of the patient. The first assistant stood on the side opposite to that of the surgeon. The retroperitoneum and right colon mesocolon were divided, exposing the ventral aspect of the superior mesenteric vein. The ileocolic vessels, right colic vessels and midcolic vessels were identified in that order. The terminal ileum, cecum and ascending colon were mobilized up to the hepatic flexure, while the duodenum and right ureter were being protected. In the left hemicolectomy, using the medial approach, the inferior mesenteric artery was identified. An anastomosis was made by a small laparotomy or by endoscopic intraluminal anastomosis.

### Follow-up

One month after surgery and every 3 months thereafter, a physical examination was performed and levels of laboratory markers, such as serum carcinoembryonic antigen and carbohydrate antigen 19.9, were assessed. At each patient visit, symptoms were recorded and wound scars were examined. Either ultrasonography or CT scans of the abdomen, in addition to chest X-rays, were performed every 6 months, and a total colonoscopy was performed every year. All patients were followed-up subsequent to being discharged from the hospital. Survival was calculated in months from the date of diagnosis to the date of mortality or to the date of the last visit to the outpatient clinic. For patients who did not visit the hospital, telephone interviews were performed. The last date for follow-up was April 2013. Data collected included local recurrence, distant metastasis and survival.

### Statistical analysis

All calculations were performed using SPSS software, version 17.0 (SPSS, Inc., Chicago, IL, USA). Parametric variables are expressed as the mean ± standard deviation (SD). Categorical data are presented as the frequencies and percentage and were compared by the χ^2^ test. Parametric and non-parametric continuous data are presented as the mean ± SD and evaluated by Student’s t-test and the Mann-Whitney U test, respectively. The Kaplan-Meier method was used to calculate the survival data, and differences were compared by the log-rank test. P<0.05 was considered to indicate a statistically significant difference.

## Results

### Demographic and pre-operative clinical characteristics

A total of 160 patients were enrolled and the medical records were retrospectively analyzed in this study. Of the surgeries performed during the study period, 80 cases were laparoscopic-assisted colorectal resections and 80 cases were conventional open surgeries. No statistically significant differences were found in the majority of the demographic and pre-operative clinical parameters between the two patient populations (Table I).

### Surgical procedures and pathological parameters

No statistically significant differences were found in the surgical procedures between the two groups (Table II). The resection margins were similar in the two groups and none were found to be positive for cancer cells. There were no significant differences in the number of lymph nodes sampled, the total sample length or the TNM staging (Table II). A significant difference was observed in the length of surgery between the two groups (201.7±6.91 min for laparoscopic vs. 177.2±7.2 min for open surgery; P=0.015; Table III). Moreover, a significantly lower level of blood loss was found during laparoscopic-assisted surgery compared with open surgery (P=0.002) (Table III). Only one patient (1.25%) was converted from laparoscopic-assisted to open surgery.

### Perioperative recovery

The patients who underwent the laparoscopic-assisted procedure showed a significantly faster recovery time than those who underwent open surgery, namely, less time to first passing flatus (P<0.001), first bowel movement (P=0.009), resuming a liquid food diet (P=0.015) and walking independently (P=0.006) (Table III). Compared with the patients who underwent open surgery, laparoscopic-assisted colorectal surgery notably caused less pain for patients resulting in a lower requirement for analgesics (P=0.001) and a shorter hospital recovery time (10.7±0.59 days for laparoscopic-assisted vs. 12.36±0.67 days for open surgery; P=0.007). However, laparoscopic-assisted colorectal surgery resulted in higher surgery expenditure (P=0.003) and total hospitalization costs (P<0.001) compared with open surgery (Table III). There was no statistically significant difference in post-surgical costs between the two groups (Table III).

### Complications

No significant difference was found in the number of adverse events during surgery between the laparoscopic and open surgery groups (Table IV). The majority of the intraoperative and post-operative complications were minor in the two groups and almost all were due to wound infection.

### Recurrence and survival

**Figure 1:**
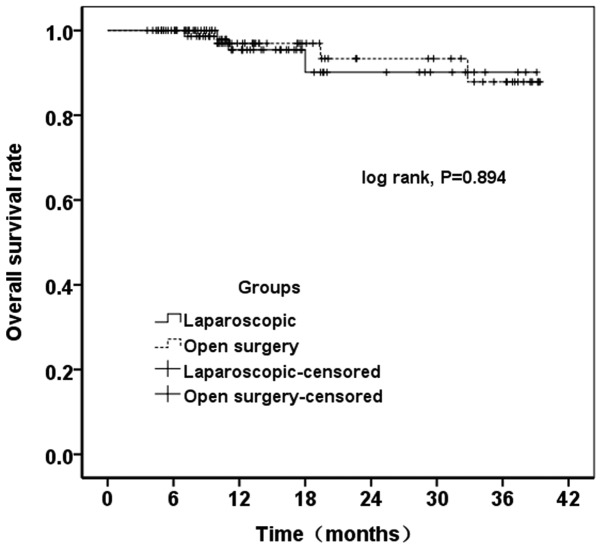
Overall survival rate of laparoscopic versus open surgery patient groups.

**Figure 2:**
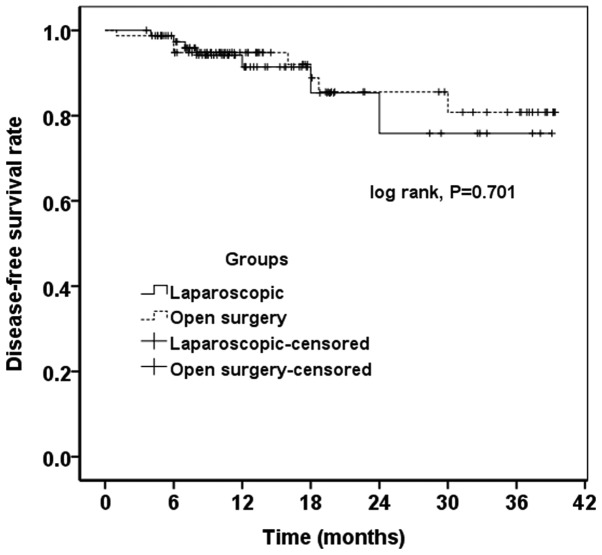
Disease-free survival rate of laparoscopic versus open surgery patient groups.

No significant difference in the rate of recurrence between the two groups was found (Table V). The mean follow-up times were 17.5 and 18.2 months in the laparoscopic and open surgery groups, respectively. According to the results of the Kaplan-Meier analysis, the laparoscopic and open surgery groups did not have significant differences in overall survival (P=0.894) (Fig. 1) and disease-free survival (P=0.701) rates (Fig. 2).

## Discussion

Since Jacobs *et al* (18) completed the first laparoscopic-assisted colectomy in the world, laparoscopic-assisted surgery for colorectal cancer has been widely performed. Over the past two decades, improvements have increasingly been made to the laparoscopic-assisted resection of colorectal cancer. However, laparoscopic-assisted colorectal surgery, which is the gold standard treatment for colorectal cancer, has controversial oncological stability. The present study compared and analyzed data on patients with colorectal carcinoma who underwent laparoscopic-assisted or conventional open surgery. The results indicated that laparoscopic-assisted surgery had the clear advantages of a minimally invasive surgery and comparable rates of recurrence and survival compared with that of conventional open surgery.

A number of previous studies (9,11,19–21) reported that patients who underwent laparoscopic-assisted colorectal cancer surgery possessed several advantages, including less bleeding, less trauma, a faster recovery of bowel function and a shorter hospital stay. In the present study, significant improvements in post-operative recovery among laparoscopic-treated patients were observed, with shorter times to first passing flatus and ambulation, earlier resumption of a liquid food diet and a shorter post-operative hospital stay. These results were consistent with a number of domestic and foreign studies (22,23). Thus, the advantages of minimally invasive surgery were confirmed.

The post-operative hospital stay for the patients who underwent the laparoscopic procedure ranged between 5 and 8 days in certain randomized controlled trials (12,24–25), which was a shorter time than the 10.7 days reported in the present study. Several confounding factors could have affected the comparison of the hospital stay between the two groups, as well as between studies. For example, certain variables, such as the pre-operative health status of the patients and chemotherapy may have extended the length of hospital stay for all patients. As pre-operative comorbidities may affect post-operative recovery, and patients could not be discharged until the end of the first regimen of post-operative chemotherapy, such covariates were examined to assess any substantial differences between the two groups.

The mean operating time of the laparoscopic procedure versus open surgery varied among studies, with certain studies reporting no differences between the two groups (11,26), and others reporting a significantly longer time for the laparoscopic procedure. This may be due to the higher complexity of technical expertise involved in such techniques (27). In the present study, a longer operating time was observed for the laparoscopic procedure compared with open surgery, and this difference was significant. Therefore, with the stabilization of the learning curve of the surgeon, the operating time may be significantly reduced in the future.

Higher treatment costs were a relative disadvantage in the laparoscopic group of the present study. Laparoscopic colorectal surgery caused higher surgery expenditure (P=0.003) and total hospitalization costs (P<0.001) compared with open surgery. Kapritsou *et al* (28) found that the surgery costs in the laparoscopic group were significantly higher than those in the open surgery group. In addition, Steele *et al* (29) reported that the total hospitalization costs in the laparoscopic group were significantly higher than those in the open surgery group. We hypothesize that the reason for the higher surgery expenditure and total hospitalization costs in laparoscopic-assisted surgery is that disposable endoscopic supplies and laparoscopic instruments are more expensive overall.

The conversion rate of the present study was 1.3%, which was notably lower than that reported in other studies, which ranged between 15 and 30% (12,25,30–32). The variation among studies may be due to the evolution of operating skills over time, thus reducing the conversion rates in the more recent studies. In addition, as the learning curve of the technique was incorporated during the study period and the skills were evolved during the conduct of the study, it is not unexpected that the number of conversions was lower in the latter phase of the present study.

The present study assessed the oncological safety by examining the post-operative results, such as the resection margin and the number of resected lymph nodes. The results indicated that the laparoscopic-assisted procedural outcomes were comparable to those achieved by open surgery. None of the resection margins were found to be positive for cancer cells, as reported in the majority of previous studies with data on resection margins (25,26,33–36). The mean number of resected lymph nodes was 11.86±1.95 and 12.24±1.17 in the patients who underwent laparoscopic-assisted and open surgery, respectively, thus confirming that there were no differences in the number of lymph nodes harvested between the two groups. These findings indicated that the oncological safety of the laparoscopic-assisted surgery in the present study was comparable to previous results (37,38).

The long-term outcomes of laparoscopic-assisted surgery for colorectal cancer from three major multicenter trials have not yet been determined (12,30,39). In the present study, the follow-up outcomes, including rates of local recurrence, distant metastasis, overall survival and disease-free survival, were assessed over 1 year, and the median follow-up time was ~17.9 months for each group. With regard to the recurrence rate, patients who underwent laparoscopic-assisted surgery displayed rates comparable to those who underwent open abdominal surgery. The study revealed that the recurrence rate for patients with colorectal cancer was lower than the prospective trials, with ~3–7% and 17–19% local and distant recurrence rates, respectively (7,13,24,38). This may be associated with the small sample size and short follow-up time. Furthermore, the follow-up time for all is ≤3 years, so the laparoscopic equipment used was relatively advanced, therefore the surgery was relatively easy to perform. However, the number of patients with recurrent colorectal cancer was similar in the laparoscopic-assisted and open surgery groups of these studies, and these results were comparable to the present study. Similar overall and disease-free survival rates in the two groups confirmed the long-term oncological safety of the laparoscopic approach compared with open surgery. The long-term follow-up results conducted in prospective studies were reviewed and the 3-year survival rates were ~85% in almost all studies (13,24), whereas in other previous studies they were significantly lower (<70%) (26). With regard to the 5-year survival rate, a certain degree of controversy has been found among different studies (data ranging between 65.3 and 77%) (13,14). The present results were consistent with those findings in which laparoscopic-assisted surgery appeared to be equivalent to the open method.

The present study was limited in that the patients were partially non-randomized into the two treatment arms. However, as there were no differences in demographic data, we suggest that this bias had a negligible affect on the results. In addition, the mean follow-up time was short, which may cause deletions of the long-term follow-up results; thus, we cannot provide a more reliable basis with regard to the long-term outcomes.

In conclusion, the present results indicated that laparoscopic-assisted surgery for colorectal cancer is a safe and feasible approach. Laparoscopic-assisted colorectal cancer surgery possessed the clear advantages of a minimally invasive surgery; however, it also had certain disadvantages, including a longer surgery time and higher surgery expenditure and hospitalization costs. Laparoscopic-assisted colorectal cancer surgery had similar rates of recurrence and survival compared with open surgery.

## Figures and Tables

**Table I:** Demographic and pre-operative clinical characteristics.

Characteristics	Laparoscopic (n=80)	Open surgery (n=80)	P-value
Gender			0.265
Male	48 (60.0)	41 (51.3)	
Female	32 (40.0)	39 (48.8)	
Age, years	64.35±1.18	65.1±1.38	0.569
BMI, kg/m^2^	26.7±4.0	27.3±4.6	0.342
Tumor location			0.205
Colon	38 (47.5)	47 (58.8)	
Rectum	42 (52.5)	33 (41.2)	
ASA classification			0.443
I	45 (56.3)	43 (53.8)	
II	23 (28.8)	19 (23.8)	
III	12 (15.0)	18 (22.5)	
Pre-operative comorbid diseases			
Hypertension	7 (8.8)	9 (11.3)	0.598
Coronary heart disease	4 (5.0)	5 (6.3)	0.732
Diabetes	11 (13.8)	13 (16.3)	0.658
Hepatic cirrhosis	0 (0.0)	1 (1.3)	0.316
Cerebral infarction	1 (1.3)	2 (2.5)	0.560
Others	2 (2.5)	3 (3.8)	0.650

Data are expressed as the number (%) or mean ± standard deviation values. BMI, body mass index; ASA, American Society of Anesthesiologists.

**Table II:** Surgical procedures and pathological parameters.

Procedure/parameter	Laparoscopic (n=80)	Open surgery (n=80)	P-value
Procedures			
Right hemicolectomy	18 (22.5)	21 (26.3)	0.416
Left hemicolectomy	5 (6.3)	12 (15.0)	
Sigmoid colectomy	15 (18.8)	14 (17.5)	
Low anterior resection	35 (43.8)	26 (32.5)	
Abdominoperineal resection	5 (6.3)	6 (7.5)	
Total colectomy	2 (2.5)	1 (1.3)	
Conversion to open surgery	1 (1.3)	-	
Tumor size, cm	4.87±0.21	5.24±0.24	0.251
Proximal margin, cm	11.04±2.2	11.12±2.7	0.721
Distal margin, cm	8.15±3.62	8.24±3.67	0.543
Total sample length, cm	24±5.76	25.19±5.91	0.522
No. of lymph nodes sampled	11.86±1.95	12.24±1.17	0.363
Positive resection margin	0 (0)	0 (0)	
TNM stage			0.715
I	16 (20.0)	15 (18.8)	
IIA	12 (15.0)	14 (17.5)	
IIB	17 (21.3)	16 (20.0)	
IIC	2 (2.5)	3 (3.8)	
IIIA	2 (2.5)	5 (6.3)	
IIIB	22 (27.5)	20 (25.0)	
IIIC	9 (11.3)	7 (8.8)	

Data are expressed as the number (%) or mean ± standard deviation values. TNM, tumor node metastasis.

**Table III:** Intraoperative data and post-operative outcomes.

Data/outcome	Laparoscopic (n=80)	Open surgery (n=80)	P-value
Surgery time, min	201.7±6.91	177.2±7.2	0.015
Blood loss, ml	97.25±9.97	221.3±37.46	0.002
Time in days to			
First passing flatus	2.34±0.12	3.80±0.17	<0.001
First bowel movement	3.43±0.28	4.87±0.18	0.009
Resume liquid food	3.66±0.15	4.34±0.19	0.015
Walk independently	1.63±0.11	2.22±0.17	0.006
Incision length, cm	5.0±0.18	19.9±0.62	<0.001
Hospital stay, days	9.7±0.59	11.36±0.67	0.007
Treatment costs			
Surgery expenditure, thousand yuan RMB	8.1±3.1	3.9±1.1	0.003
Post-surgical costs, thousand yuan RMB	9.6±3.7	10.8±6.5	0.372
Total hospitalization costs, thousand yuan RMB	48.3±10.7	26.9±7.5	<0.001

Data are expressed as the number (%) or mean ± standard deviation values. RMB, Renminbi.

**Table IV:** Intraoperative and post-operative complications for colorectal cancer.

Complications	Laparoscopic (n=80)	Open surgery (n=80)	P-value
Intraoperative complications			
Massive hemorrhage	1 (1.3)	2 (2.5)	0.560
>1,000 ml			
Organ injury	1 (1.3)	3 (3.8)	0.311
Others	2 (2.5)	1 (1.3)	0.560
Post-operative complications			
Anastomotic hemorrhage	2 (2.5)	4 (5.0)	0.405
Abdominal hemorrhage	3 (3.8)	5 (6.3)	0.468
Anastomotic stenosis	1 (1.3)	0 (0.0)	0.316
Ileus	1 (1.3)	2 (2.5)	0.560
Intestinal adhesion	1 (1.3)	1 (1.3)	1.000
Enteroparalysis	0 (0.0)	1 (1.3)	0.316
Wound infection	3 (3.8)	10 (12.5)	0.053
Lung infection	2 (2.5)	4 (5.0)	0.405
Dysuria	0 (0.0)	1 (1.3)	0.316

Data are expressed as the number (%).

**Table V:** Local recurrence and distant metastasis.

Recurrence/metastasis	Laparoscopic (n=80)	Open surgery (n=80)	P-value
Local recurrence			
Anastomotic recurrence	2 (2.5)	1 (1.3)	0.560
Pelvic recurrence	1 (1.3)	2 (2.5)	0.560
Perineal recurrence	1 (1.3)	0 (0.0)	0.316
Total	4 (5.0)	3 (3.8)	0.699
Distant metastases			
Liver metastases	1 (1.3)	2 (2.5)	0.560
Lung metastases	1 (1.3)	2 (2.5)	0.560
Extensive abdominal metastasis	1 (1.3)	0 (0.0)	0.316
Total	3 (3.8)	4 (5.0)	0.699

Data are expressed as the number (%).
